# Metabolic Profile of Male Cannabis Users and Estimation of Candidate Biomarkers

**DOI:** 10.1021/acs.chemrestox.5c00274

**Published:** 2025-10-10

**Authors:** Esin Oz, Merve Kasikci, İbrahim Celik, Mukaddes Gurler

**Affiliations:** † Department of Medical Biochemistry, Faculty of Medicine, 37515 Hacettepe University, Ankara 06100, Turkey; ‡ Department of Biostatistics, Faculty of Medicine, Hacettepe University, Ankara 06100, Turkey; § Faculty of Medicine, Hacettepe University, Ankara 06100, Turkey; ∥ Department of Forensic Medicine Forensic Toxicology Laboratory, Hacettepe University, Ankara 06100, Turkey

## Abstract

Tetrahydrocannabinol (THC), the primary psychoactive compound of cannabis, is the most widely abused substance worldwide, with an annual prevalence of 4.3% of adults and 5.3% of the 15–16 year-old population estimated as of 2022. THC has both acute and chronic effects through the dopaminergic and endocannabinoid systems. This study was conducted to better understand the metabolites and metabolic pathways in biological systems affected by cannabis, which may help find practical diagnostic and treatment approaches for people with cannabis dependence in the future. Metabolomic analysis of urine samples was performed using gas chromatography–mass spectrometry (GC–MS). MetaboAnalyst software was used to determine sample metabolite profiles, which were then subjected to multivariate statistical analysis. From data of over 200 metabolites in each sample of cannabis users, 92 metabolites with a *p*-value of less than 0.05 were selected for further analyses, of which 38 showed a decrease and 54 showed an increase compared to the nonuser group. Based on 43 metabolites (VIP > 1), subjected to MetaboAnalyst and CPDB, amino acid metabolism (especially arginine, methionine, and cysteine), vitamin metabolism (particularly biotin), and the urea cycle were the primarily affected metabolic pathways. The AUC values of the four metabolites (salsoline, 6-thiourate, procollagen 5-hydroxy- *l* -lysine, and biotin) with the highest VIP scores were between 0.93 and 0.98, with no significant difference. Metabolites with high VIP scores hold promise as biomarker candidates for identifying cannabis users, and the prominent pathways provide new insights into the understanding of the metabolic effects of cannabis.

## Graphical abstract

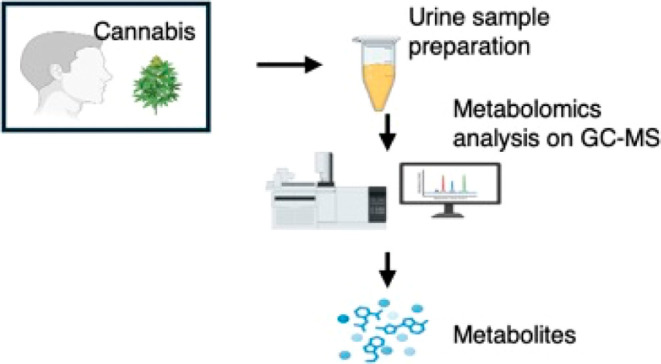

## 1 Introduction

Cannabis is one of the most widely used psychoactive substances worldwide for recreational purposes, especially prevalent in North America and affluent nations in Europe and Oceania. However, its use is increasing in low- and middle-income countries (LMICs), while it remains comparatively low in Asia.^1^ In 2021, it was estimated that 219 million people, or 4.3% of the global adult population (ages 15–64), had used cannabis.^2^ Among cannabinoids, the most well known for their pharmacological effects are psychoactive Δ9-tetrahydrocannabinol (THC) and cannabidiol (CBD). These compounds are first generated as acidic precursors rather than as direct products in the metabolic pathway.^3^ THC is primarily responsible for the psychoactive and therapeutic effects of cannabis. It induces euphoria, enhances sensory perception, and provides antinociceptive effects. THC also has acute and chronic effects on the dopaminergic and endocannabinoid systems.^4^ THC mainly produces its impact as a partial agonist on the commonly found Gi/Go protein-coupled cannabinoid receptors 1 (CB1) and 2 (CB2). The psychomimetic effects of THC are influenced by CB1.^4,5^ CB1 receptors are abundantly expressed in the central nervous system (CNS) and peripheral nervous system as well as in non-neural tissues. In contrast, CB2 receptors are primarily located on immune and glial cells.

When smoking THC, its main psychoactive component is quickly absorbed from the lungs into the bloodstream.^6^ THC is broken down through oxidation into 11-hydroxy-D9-tetrahydrocannabinol (11-OH-THC) and 11-nor-9-carboxy-D9-tetrahydrocannabinol (THC-COOH). THC is mainly stored in fat cells and continues to passively enter the blood over several days after cannabis use. THC-COOH levels rise slowly as THC is metabolized, and stay stable and detectable for several days to weeks.^7^ Thus, the most established urinary biomarker for cannabis use is THC-COOH. Urinary cannabinoids are commonly utilized as biomarkers for the detection of cannabis and synthetic cannabinoid use. Identifying specific metabolites in urine is essential for monitoring drug abuse, conducting forensic investigations, and understanding drug metabolism. Cannabinoid detection times in urine depend on pharmacological factors (e.g., drug dose, route of administration, frequency of use, and individual absorption), and methodological issues like analytes evaluated, matrix, hydrolysis type, cutoff used, and method sensitivity.^8^

Metabolomics examines the collection of small molecules that serve as structural, signaling, and metabolic components within a biological environment. This environment includes cells, tissues, fluids, and complex biological microenvironments like atherosclerotic plaques and tumors.^9^ Urine is an ideal biofluid for metabolomic epidemiology and population-based molecular phenotyping due to its noninvasive and convenient collection. Its chemical composition provides a comprehensive overview of systemic metabolism, offering valuable insights into the physiological state of the organism.^10,11^ Recently, metabolite analysis has gained significant attention to enhance our understanding of disease mechanisms and the effects of pharmacological treatments on various physiological functions. Many clinical research fields utilize metabolomics analysis to strengthen the knowledge of biochemical mechanisms, aiming to improve diagnosis, personalize medicine, and discover new therapeutic targets and concepts.^12^ Complementary analytical technologies frequently used in metabolomic analysis include nuclear magnetic resonance spectroscopy, liquid chromatography–mass spectrometry (LC–MS), and gas chromatography–mass spectrometry (GC–MS).^13^ Gas chromatography (GC), using high-resolution capillary columns paired with mass spectrometry (MS) detection, acts as a strong platform for metabolome analysis. GC–MS is especially well suited for volatile and thermally stable analytes.^14^ In the discovery phase of research, such as biomarker identification for medical diagnostics, the nontargeted approach with GC–MS is commonly used.

Metabolomic studies of cannabis users (CU) are scarce. A recent study used twins and examined plasma metabolites, proteins, and lipids, which were then compared to plasma THC-COOH levels. They found that THC-COOH levels were associated with immune system-related pathways.^7^ Another study found that plasma phosphoric acid, glycine, malonic acid, and 9.12-octadecanoic acid levels were higher in CU than in nonusers (NU).^15^

In the literature, the lack of metabolic studies in the urine of CU is striking. In this study, we performed a metabolomic evaluation in urine samples of CU to better understand the metabolites and metabolic pathways in biological systems influenced by cannabis, which may assist in finding further practical diagnostic and treatment approaches for individuals with cannabis dependence in the future.

## 2 Materials and Methods

### 2.1 Chemicals

Urease, pentadecanoic acid, and methoxyamine hydrochloride (Meox, 98%) were purchased from Sigma–Merk (Manheim, Germany). N, O–Bis­(trimethylsilyl)­trifluoroacetamide with trimethylchlorosilane (BSTFA + 1%TMCS) was obtained from Sigma as ready-to-use ampules. All other chemicals were purchased from Sigma–Merck.

### 2.2 Sample Information

Urine samples sent for drug confirmation from different screening laboratories were used in this study. The study consisted of 50 samples from male subjects in total, with 30 being CU and 20 being NU. The mean age of CU was 33.87 ± 7.13 years whereas that of NU was 33.25 ± 9.54. There was no statistically significant difference in age means between these groups based on the independent sample *t*-test (*t* = 0.261, *p*-value = 0.795). CU used cannabis daily for at least 1–3 months. Their cannabis metabolite (THC-COOH) levels were detected between 16 and 771 ng/mL by using a validated LC–MS/MS method with a cutoff level of 5 ng/mL.

### 2.3 Sample Preparation for GC–MS Analysis

Urine sample preparation involved a two-step derivatization process comprising methoxymation and silylation reactions. Urine samples were thawed at room temperature for 1 h and vortex-mixed. Urease buffer was prepared at 15 U in ultrapure water. Urine samples (200 μL) were placed in centrifuge tubes. Then, 50 μL of urease solution (15U) was added to each tube to decompose and eliminate the excess urea. The samples were incubated at 37 °C for 30 min. Next, 800 μL of cold methanol (cooled at −80 °C for 30 min) and 10 μL of pentadecanoic acid (used as an internal standard at 1 mg/mL in methanol) were added to each tube. The samples were then vortexed for 5 min and centrifuged at 3000 rpm for 15 min at 4 °C. 200 μL of the supernatant was transferred into glass inserts in GC vials and then evaporated to dryness at 30 °C. The residues were methoxyaminated with 30 μL of methoxyamine hydrochloride (15 mg/mL in pyridine) by incubating for 16 h at room temperature in the dark. Then, 30 μL of BSTFA with 1% TMCS was added to each sample, vortex-mixed for 5 min, and derivatized by incubating at 70 °C for 1 h. Before GC–MS analysis, 70 μL of heptane was added, and vortex-mixing for 10 min was performed.

The quality control samples were prepared to control system stability and guarantee the method’s reproducibility. They consisted of pooled biological samples prepared by combining equal volumes (50 μL) from each urine sample analyzed. The QC samples were prepared by using the same procedure described above and analyzed along with other samples. Heptane blank samples were analyzed at the start and end of the sequence runs.^16^

### 2.4 Instrumental Features

Metabolomics analysis were performed by using an Agilent 7890B GC-5977 MS with electrospray ionization (ESI) at 70 eV. An HP-5 ms column (86% dimethylpolysiloxane and 14% Cyanopropylphenyl, 30 m × 0.25 mm) with a 5 m guard column was used for chromatographic separation. Ionized samples were injected into the MS with a volume of 1 μL. The inlet temperature was 290 °C and was held for 1 min in a splitless mode at 180 kPa. The helium gas was flowed at 1.0 mL/min through the column. The initial oven temperature was set to 45 °C, ramped to 180 °C with a rate of 9 °C/min in 2 min, kept constant for 5 min, then increased to 220 °C (40 °C/min), and held stable for 5 min. Afterward, with a rate of 40 °C/min, the temperature was first increased to 240 °C, then to 280 °C, and held constant for 11.5 and 2 min, respectively. The transfer interface and ion source were kept at 250 °C, while the quadrupole was set to 130 °C. The MS detector functioned within a range of 30–550 amu during the analysis.

### 2.5 Preprocessing

The metabolomics data was obtained using the GC–MS library (NIST MS Library). An XCMS online tool^17^ was used for preprocessing based on GC/Single Quad (centWave) parameters. Concentration values for the metabolites were obtained. The metabolomic data was analyzed with AMDIS (automated mass spectral deconvolution and identification system), resulting in a metabolite peak data matrix that includes SpectConnect.^18^ The metabolomic data matrices were uploaded to MetaboAnalyst4.0 ^19^ and normalized using the sum area-mean centering. Metabolite traits with over 50% missing values were removed from the data matrix. KEGG IDs of the identified metabolites were obtained. Since metabolites have a wide range of values, normalization was applied to the data set. Normalization by the median was selected as the normalization method for its robustness to outliers. Logarithmic transformations were also performed to obtain less skewed distributions before the univariate analysis of all metabolites.

### 2.6 Data Analysis

An independent sample *t*-test was employed to assess whether there was a statistical difference between CU and NU. The statistical significance level (α) was taken as 0.05. Testing a large number of metabolites may increase the type *I* error rate. Therefore, in addition to the raw *p*-values, we also reported the adjusted *p*-values, also known as the false discovery rate (FDR). Following the evaluation of the metabolites individually, multivariable analyses were conducted to assess the effects of the variables together. Partial least-squares discriminant analysis (OPLS-DA) was performed to visualize how CU and NU were distinguished by using significant metabolites together. Also, variable importance in projection (VIP) scores were obtained to assess the extent of metabolites. Metabolites with VIP scores above 1 were considered significant. To evaluate the validity of the OPLS-DA model, a permutation test with 2000 iterations was applied. Permutation test results were examined using *R* ^2^, which measures the explanatory ability of the model, and *Q* ^2^, which is an indicator of the consistency of results between the original and cross-validation data. In addition, principal component analysis (PCA) was also performed to observe metabolic differences between CU and NU in an unsupervised manner.

Classification models were developed to evaluate the performance of important metabolites in classifying CU and NU. As the classification algorithm, the elastic-net regularized generalized linear model was used. This algorithm is based on logistic regression and uses penalty parameters in the Ridge and Lasso regression methods. A 5-fold cross-validation method was used for model validation. A receiver operating characteristic (ROC) curve was drawn for the models. Area under the ROC curve (AUC) was used as the performance measure.

Metabolite sets based on KEGG metabolic pathways were obtained through enrichment analysis by using the MetaboAnalyst tool. To enhance the biological interpretation, the consensus pathway database (CPDB) tool was also used to perform pathway enrichment analysis. Both tools employ a hypergeometric test to determine whether a particular group of metabolites was more overrepresented in the given list of compounds than would be expected by chance. While MetaboAnalyst restricts its analysis to a single database, CPDB integrates information from multiple databases. Since these tools rely on different databases and versions, both were utilized to achieve a more detailed and robust enrichment analysis. MetaboAnalyst software was used for univariate analysis, OPLS-DA, and PCA. The caret *R* package^20^ was used for classification analyses. The pROC *R* package obtained the ROC curve plot and AUC values.^21^

## 3 Results

Metabolite profiles of all urine samples, including CU (*n* = 30) and NU (*n* = 20), were obtained by GC–MS, and the results were subjected to multiple statistical analyses. After calculating *p*-values, fold change values, and regulatory status, statistically significant metabolites were identified in urine samples (excluding unknown metabolites-UN). Among more than 200 metabolites found in each cannabis user sample, 92 metabolites (Table S1) with *p*-values less than 0.05 were selected for further analysis, of which 38 showed a decrease and 54 showed an increase compared to the NU group.

OPLS-DA was examined to visualize how significant metabolites distinguished CU and NU. One participant in the CU group was similar to that in the NU group in terms of the metabolite profile. With this exception, the groups were different from each other (Figure 1).

**Figure 1:**
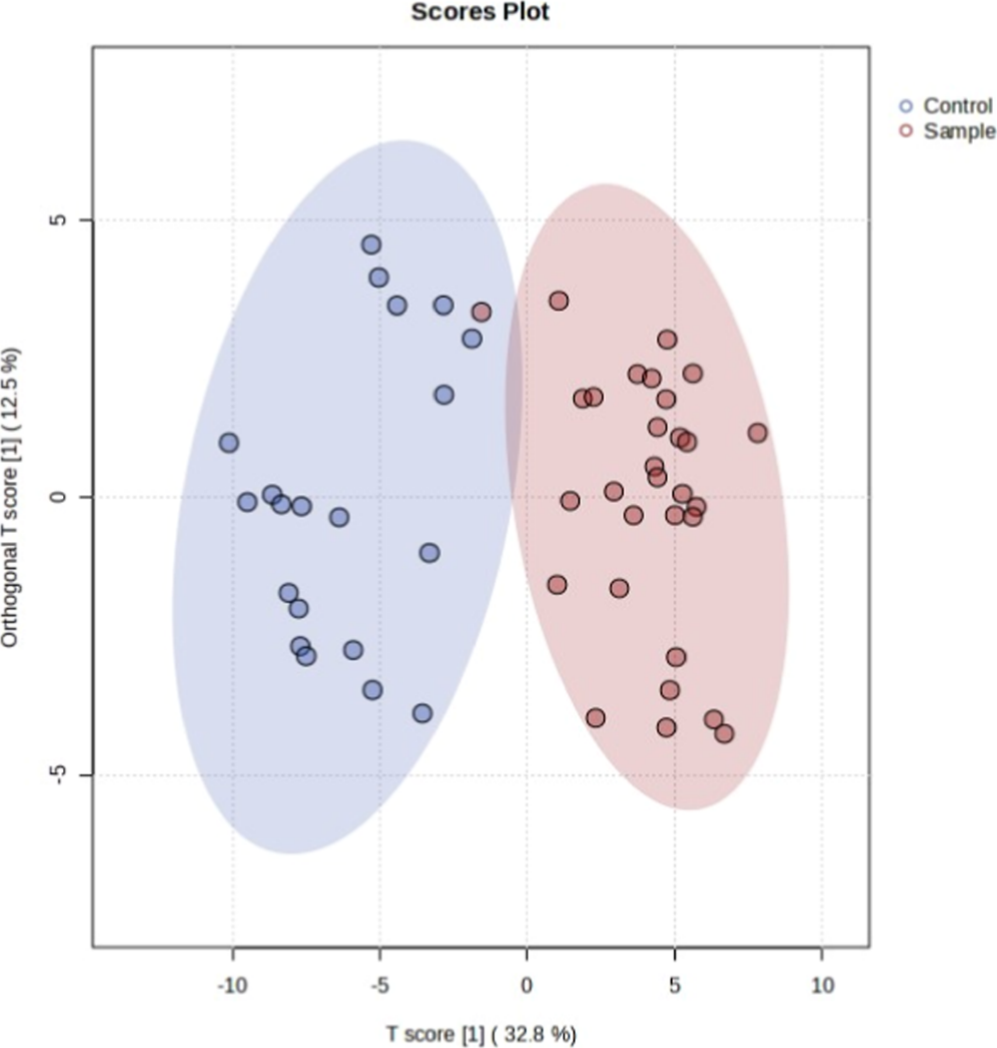
OPLS-DA two-dimensional score plots in comparison to CU and NU.

The permutation test yielded a statistically significant (*p* < 0.001) *R* ^2^ value of 0.847 and a *Q* ^2^ value of 0.773 (Figure 2), stating that the model’s validity was at an acceptable level.

**Figure 2:**
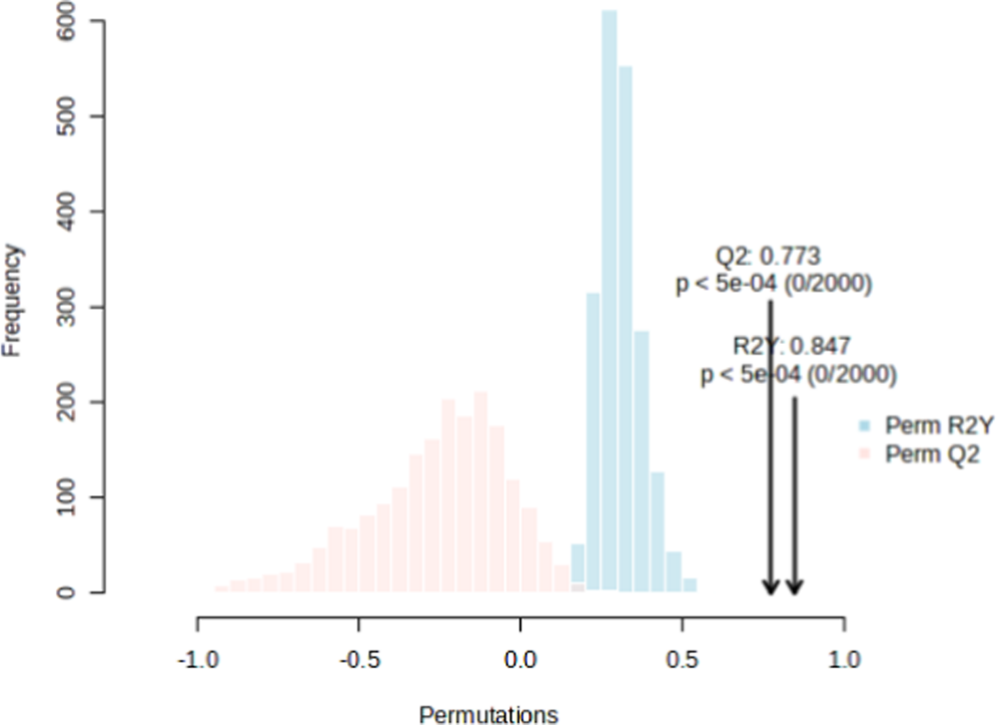
Permutation test result for OPLS-DA model. *R* ^2^ reflects the variance in the data explained by the model and indicates the quality of the fit. *Q* ^2^ shows the variance that the model can predict and indicates its predictive power.

The metabolites responsible for differentiation in the OPLS-DA graph are shown in VIP plots. The metabolite with the highest VIP value in the graph distinguishes the groups most effectively and shows the most significant statistical variation. Metabolites (43 of 92) that were found to be significant (VIP > 1) are presented in Figure 3. A table showing the VIP scores of all 92 metabolites has been presented in the supplement (Table S2).

**Figure 3:**
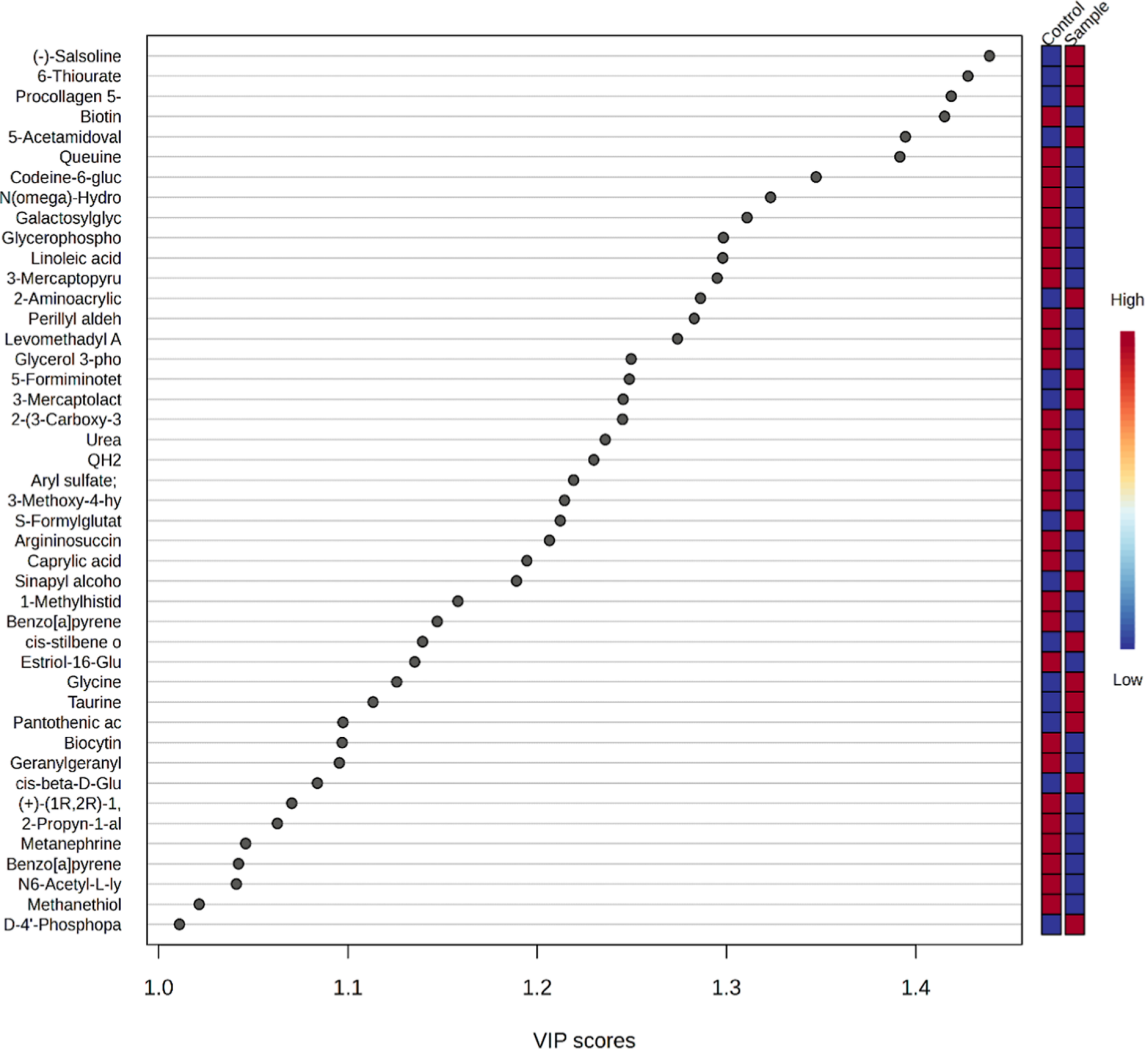
Metabolites with a VIP score greater than 1.

The heat map diagram showed that there was a significant difference in metabolite profiles between NU (control) and CU (sample; Figure 4).

**Figure 4:**
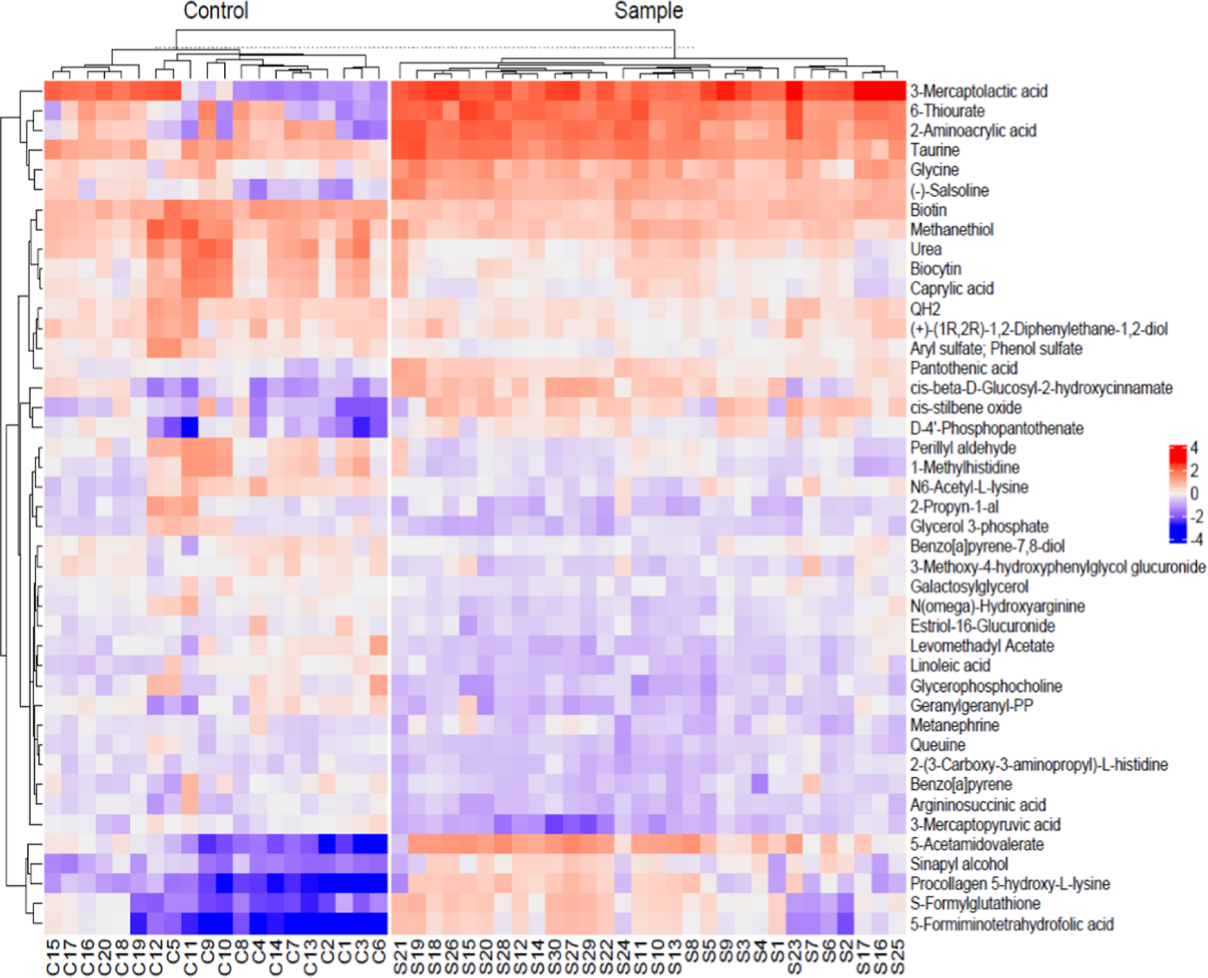
Heatmap for metabolites with VIP scores greater than 1 (C1:1.control = nonusers, S1 = 1.sample = CU).

OPLS-DA is a classification-based approach, in which group labels are included in the analysis. In addition to this supervised approach, PCA was also performed to assess whether the groups exhibit separation without using prior group information. According to Figure 5, the CU and NU groups are visibly separated in the PCA space. The NU group exhibits a broader and more dispersed distribution, whereas the CU group forms a tighter cluster. This indicates that the NU group is more heterogeneous compared to the CU group. Additionally, a few NU samples show metabolomic profiles that are closer to those of the CU group than to those of the main NU cluster.

**Figure 5:**
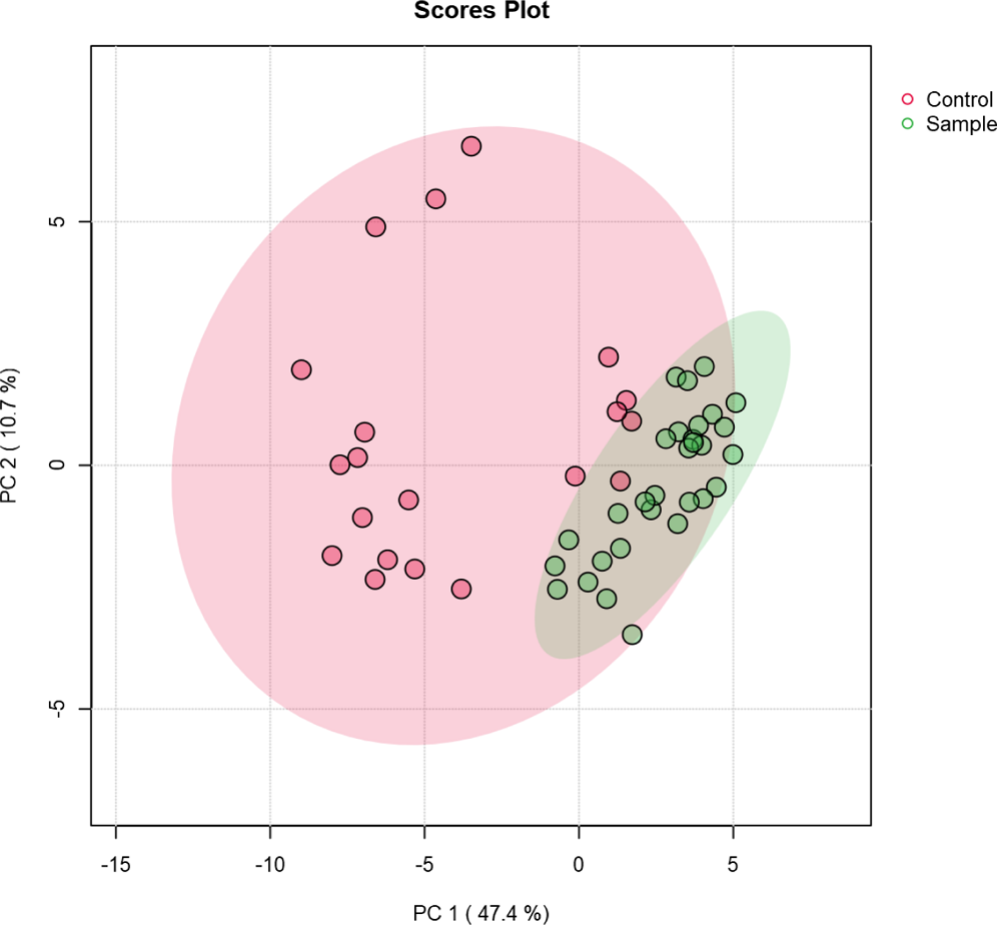
PCA two-dimensional score plots in comparison to CU and NU.

The MetaboAnalyst program was used to illustrate the pathways associated with metabolites (*n* = 43) exhibiting significant changes (*p* < 0.05) (Figure 6). The most affected pathways identified included biotin metabolism, arginine biosynthesis, fatty acid metabolism, taurine, and sulfur metabolism and the one-carbon pool by folate. Pathway enrichment analysis was also conducted on CPDB since this software simultaneously considers several databases in addition to KEGG. The results of enrichment analysis for MetaboAnalyst and CPDB are provided in the supplement (Tables S3 and S4). When both tools are considered, amino acid metabolism (especially arginine, methionine, and cysteine), vitamin metabolism (especially biotin, pantothenic acid, coenzyme *A*, and folate), and the urea cycle are the primarily affected metabolic pathways.

**Figure 6:**
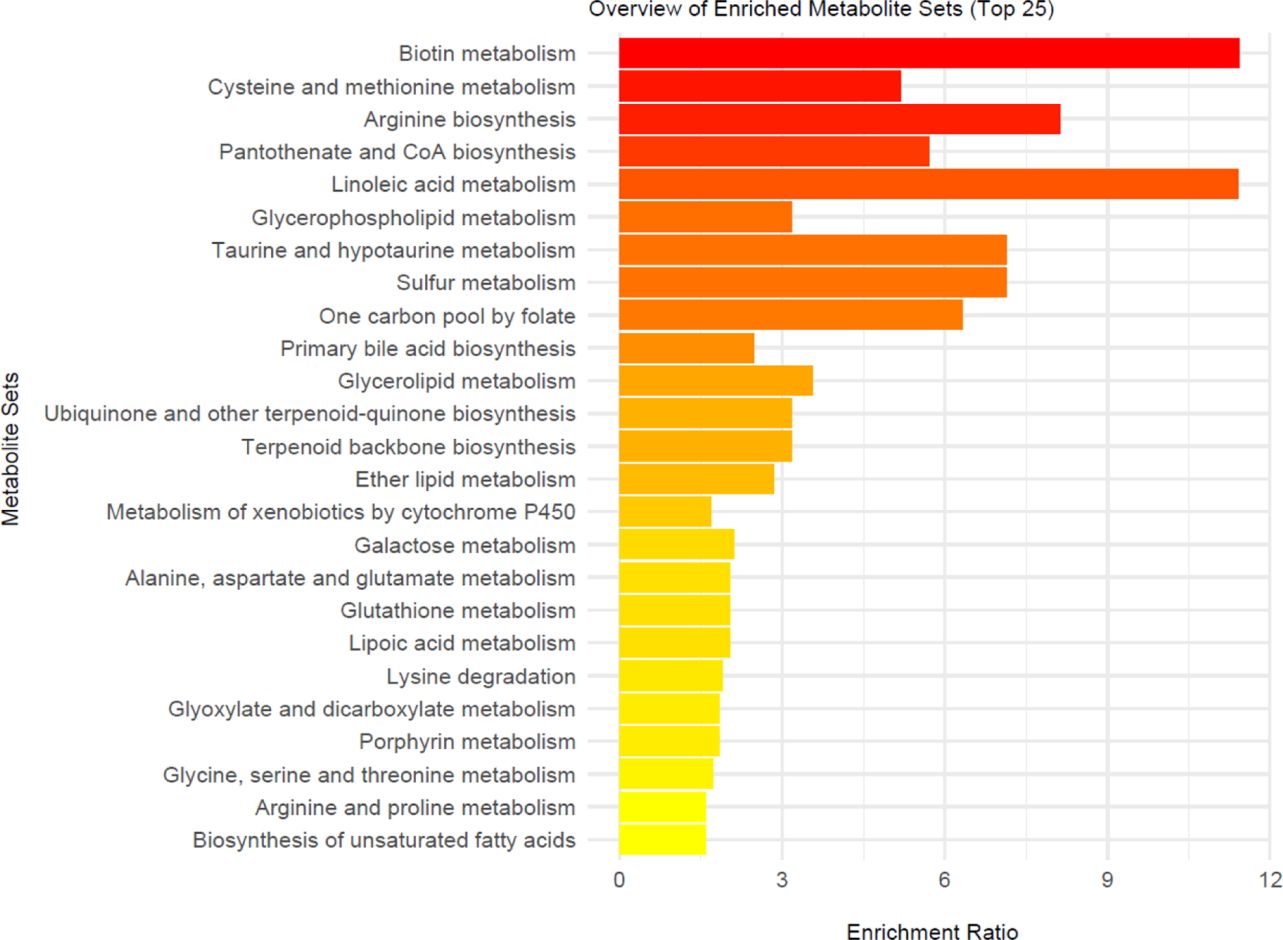
Metabolic pathways associated with the 43 metabolites that have a VIP score above 1.

Three different models based on different metabolite sets were obtained in the classification analysis. The first model includes the metabolite (Salsoline) with the highest VIP score. The second model comprises the first four metabolites with the highest VIP scores (Salsoline, 6-Thiourate, Procollagen 5-hydroxy- *l* -lysine, and Biotin). The last (third) model contains 43 metabolites with a VIP score greater than 1. ROC curves of the models are presented in Figure 7. The AUC values of the three models were found to be 0.927, 0.971, and 0.984, respectively, and were compared in pairs using the DeLong test. There were no statistically significant differences between the models by means of the AUC values (*p* values were 0.74, 0.70, and 0.68 for the model 1–2 comparison, model 1–3 comparison, and model 2–3 comparison, respectively).

**Figure 7:**
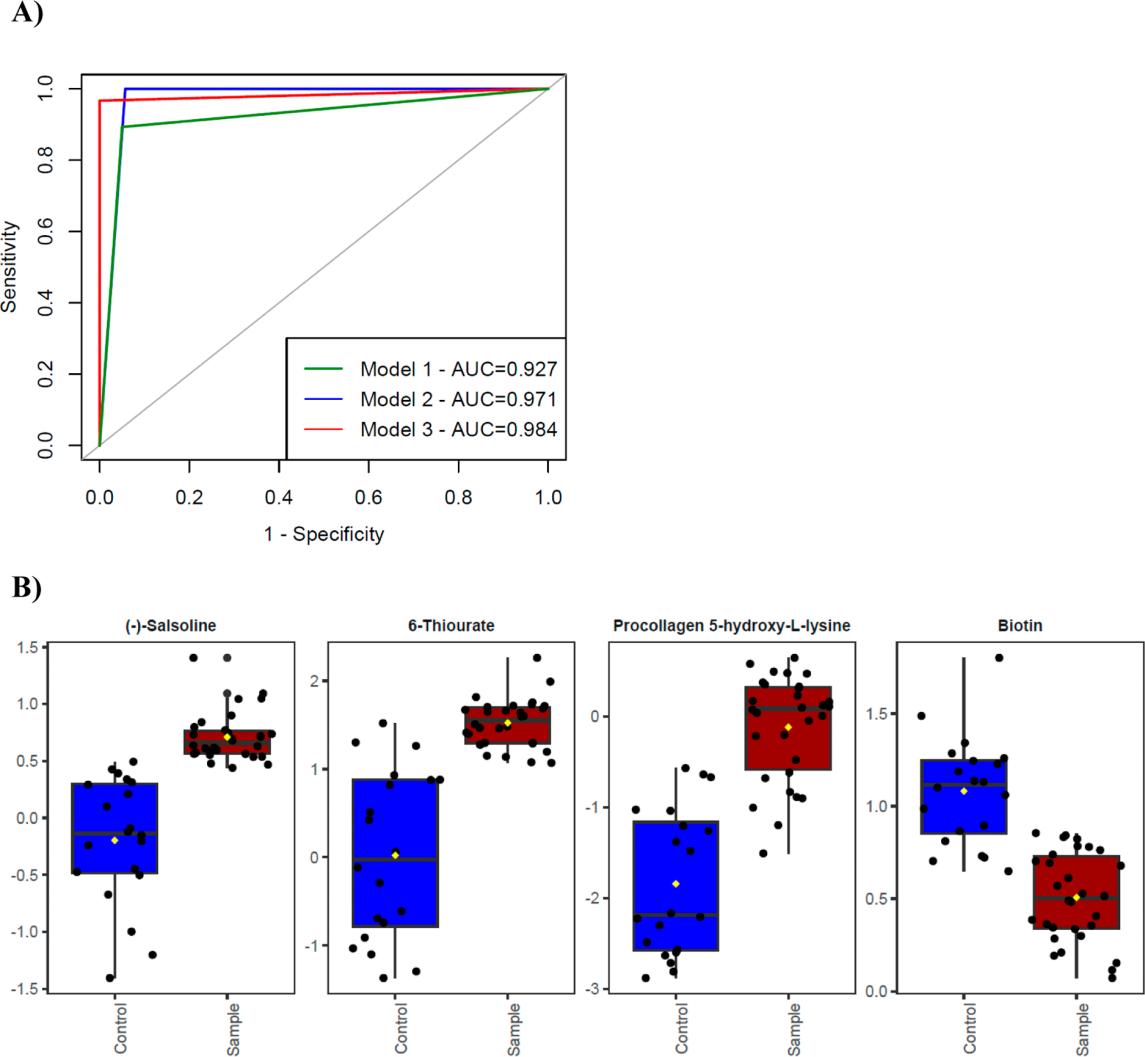
A) ROC curves for three models. (B) Box plots for the top four critical metabolites based on VIP scores. The *y*-axis represents median-normalized and log10-transformed metabolite intensities for each group.

## 4 Discussion

As cannabis becomes increasingly available and regulated, research on it is steadily gaining momentum. This study aimed to better understand the metabolites and metabolic pathways in biological systems affected by cannabis. When we evaluated over 200 metabolites in each sample from CU obtained through metabolome analysis, we identified 92 metabolites with a *p*-value of less than 0.05. By comparing the results of the CU and NU, we found a statistically significant difference between the groups (*p* < 0.05). Compared to the non-CU, 38 metabolites showed a decrease, while 54 showed an increase. Since 43 of the 92 metabolites had a VIP score above 1, further analysis was performed on these metabolites. The most affected metabolic pathways in CU were shown to be biotin metabolism, arginine biosynthesis, linoleic acid metabolism, taurine and sulfur metabolism, and the one-carbon pool by folate based on the MetaboAnalyst database. Although 43 metabolites show promise as candidate markers for distinguishing between controls and CU, it might be sufficient to choose one or some of them for further research, as ROC analysis did not show any statistical difference between the three models containing one, two, or 43 metabolites. When CPDB was used as a second tool in the enrichment analysis, biotin metabolism was found to be the most altered pathway in CU, along with arginine, cysteine, methionine, pantothenate, coenzyme A, and folate metabolism, which is mostly compatible with the MetaboAnalyst results.

Biotin, a water-soluble vitamin in all organisms, is a cofactor for biotin-dependent carboxylases. Its role in these carboxylases is to facilitate the transfer of carboxyl groups between donor and acceptor molecules during carboxylation reactions. Biotin is covalently attached to these proteins through the action of biotin ligases, referred to as BirA protein in prokaryotes and as holocarboxylase synthetase in eukaryotes.^22^ The known function of biotin in human cells is to act as a cofactor for five biotin-dependent carboxylases, which include pyruvate carboxylase, propionyl-CoA carboxylase, methylcrotonyl-CoA carboxylase, acetyl-CoA carboxylase 1 (ACC-1), and acetyl-CoA carboxylase 2 (ACC-2). These enzymes catalyze key reactions in gluconeogenesis, fatty acid synthesis, and amino acid catabolism. The metabolic and gene regulatory pathways of biotin focus on supplying functional carboxylases and enhancing the tissue-specific use of biotin while protecting brain metabolism and function during malnutrition and starvation.^23^ We observed a decrease in biotin levels in CU compared to those in NU, consistent with the alteration in metabolic pathways related to biotin.

Fatty acids are essential energy sources and critical components of membrane lipids. They also act as cellular signaling molecules that significantly influence the development of metabolic syndrome. ACC-1 and ACC-2 catalyze the synthesis of malonyl-CoA, the substrate for fatty acid synthesis, while also regulating fatty acid oxidation.^24^ Cannabinoids act as agonists for cannabis receptors CB1 and CB2, which are components of the endogenous endocannabinoid system. These receptors are in the central nervous system and periphery, particularly on immune and adipose cells. Endogenous cannabinoids, a bioactive lipid mediator, regulate food intake, peripheral energy metabolism, and lipid metabolism within adipose tissues.^25^ A study by Cisbani and colleagues examined serum fatty acid composition in a cohort of young, long-term CU compared to a group of non-CU. They found that serum levels of certain saturated and monounsaturated fatty acids, including palmitic, palmitoleic, and oleic acids, were higher in CU than in NU, consistent with the current study performed in urine samples. They also demonstrated that the endocannabinoid 2-arachidonoylglycerol is elevated in CU and may contribute to lipogenesis by activating CB1.^26^ THC has been shown to affect glutamate levels, and increased glutamate has been linked to higher oxidative stress and inflammation, both of which are associated with depression and other psychiatric conditions.^27,28^ Compatible with these findings, we found that glutamate and glutathione metabolism were significantly altered in CU compared to those in controls.

Another study by Hinckley et al. examined the molecular pathways influenced by cannabis use in humans via plasma. Their findings showed correlations involving a THC metabolite, THC-COOH, 13 proteins, three metabolites, and two lipids; taurine was one of those metabolites,^7^ as observed in our study. THC has recently been shown to interact with the nuclear factor kappa-light-chain enhancer of activated *B* cell (NF-κB) signaling pathway, which is linked to taurine that plays a role in brain development and neuroinflammation.^29^ Taurine (2-aminoethanesulfonic acid or tauric acid) is a nonprotein amino acid in various animal tissues, particularly in the brain, heart, and skeletal muscles. It also naturally occurs in foods, such as meat, fish, dairy products, and energy drinks. In humans, taurine is synthesized in the liver primarily through the cysteine sulfinic pathway.^30^ At the cellular level, it is mainly present in the intracellular fluid of various tissues, where it plays a crucial role in numerous physiological functions. As an osmolyte, taurine regulates cell volume and maintains cellular integrity.^31^ In the liver, it conjugates with bile acids to produce bile salts, which aid in fat digestion and absorption in the intestines, processes vital for lipid metabolism and the uptake of fat-soluble vitamins.^32^ Taurine also plays a role in calcium (Ca^2+^) signaling, modulates ion channels, and facilitates neurotransmission, thereby influencing the neural excitability and synaptic activity. Furthermore, it has significant antioxidant properties that protect cells from oxidative and nitrosative stress by neutralizing free radicals and reactive oxygen species.^30,33^ Few studies have linked taurine to cannabis use. Newman and colleagues demonstrated a potential relationship between cannabis use and taurine.^29^ We observed a significant change in taurine (increase) and related pathways in CU.

A distinguished study conducted proteomic research on urine samples from regular CU using liquid chromatography–tandem MS, revealing that 19 peptides were significantly altered in CU, along with changes in pathways related to immunity and carbohydrate metabolism.^34^ Five proteins (pancreatic ribonuclease, cubilin, filamin *B*, alpha-2-HS-glycoprotein, and serotransferrin) were found to be downregulated in CU, suggesting that this may be due to the antineoplastic effect of cannabis. We performed a metabolomics study using GC–MS on urine samples of CU and identified metabolic pathways and metabolites affected by cannabis differently from them (Figures 3–5).

Metabolomics studies identify metabolites associated with metabolic pathways (protein, carbohydrate, lipid, etc.), which have been effectively used as a biomarker discovery tool for the early diagnosis of diseases. In the current metabolomics study, the 43 metabolites with a VIP score above 1 seem to be more useful in distinguishing CU from NU. Among these, four metabolites (salsoline, 6-thiorate, procollagen 5-hydroxy- *l* -lysine, and biotin) with the highest VIP scores (VIP > 1.4) and AUC values between 0.93 and 0.98 might be selected as biomarker candidates.

Salsoline, a tetrahydroisoquinoline alkaloid derived from Salsola plants, is recognized for its various biological activities and antiviral effects against influenza *A* and *B* viruses.^35^ Salsoline is a monomethylated metabolite of salsolinol. Both salsolinol and its *O*-methylated derivative, salsoline, are naturally occurring compounds derived from dopamine. These compounds have been examined for their potential roles in neurochemical processes, especially concerning neurodegenerative diseases and addiction.^36^

6-Thiourate, or 6-thiouric acid, is classified as a purinone compound. It is a byproduct of the oxidation of 6-mercaptopurine (6-MP) catalyzed by xanthine oxidase. This metabolite plays a role in the metabolism of 6-MP, which can be inhibited by allopurinol, resulting in an increased production of the therapeutically active metabolite 6-thioguanine.^37^ The relationship between 6-thiourate and cannabis appears to be difficult to explain in terms of their metabolic interaction, and further research should be performed to clarify this issue.

Procollagen peptides are markers of matrix turnover. Procollagen type III amino-terminal peptide levels may reflect the fibrosis stage in chronic viral hepatitis.^38^ Increased levels of collagen propeptides have been associated with lung, liver, and heart fibrosis, severe sepsis, tuberculosis, alveolitis, Crohn’s disease, and scleroderma.^39^ High *N*-terminal procollagen peptide levels may indicate bone loss, fracture risk, and cancerous bone metastasis. Collagens with hydroxylysine-based cross-links are found primarily in cartilage, bone, ligaments, tendons, internal connective tissues, and embryonic skin.^40^ Heavy cannabis use has been associated with low bone mineral density, high bone turnover, and increased fracture risk.^41^ The increase of procollagen 5-hydroxy- *l* -lysine in our study shows the effect of cannabis on matrix metabolism.

Among the four metabolites that serve as potential candidate biomarkers, biotin may be the most privileged molecule in distinguishing CU from NU, as biotin tests are commercially available and could be used in the confirmation analysis. As previously described, biotin is an essential coenzyme involved in metabolic pathways that include lipid, amino acid, and carbohydrate metabolism. Our findings suggest that biotin levels decrease with cannabis use.

### 4.1 Limitations of the Study

The sample size is small and consists only of male participants. There is insufficient data on the participant’s history of cannabis use; the duration and dosage of drug use should be considered as factors that may influence metabolites. Also, the lack of information regarding the sampling time may affect the assessment of the metabolite profile. Additionally, there is no data on participants’ dietary habits, medications, or pre-existing diseases, which are important factors that can alter metabolism.

Unfortunately, confirmation of the candidate biomarkers obtained in this study could not be performed due to the limited budget and lack of reference standard materials required for method establishment and validation for the targeted LC–MS/MS analysis.

## 5 Conclusion

In summary, based on the results obtained, any of the 43 metabolites with a VIP score above 1 may be useful in distinguishing CU from NU. Among these metabolites, salsoline, procollagen-5-hydroxy lysine, and biotin could serve as candidate biomarkers for identifying CU. We think that our study contributes new information to the literature on the metabolic effects and promising biomarkers of cannabis use. However, given the study’s limitations and the need to confirm the current preliminary findings by performing quantitative targeted analysis, further controlled studies should be conducted with larger sample sizes and different genders. In particular, the specificity of candidate biomarkers, especially biotin, for CU should be tested.

## Supplementary Material



## Data Availability

The data sets generated and analyzed during the current study are available from the corresponding author upon request.

## Notes

*P*-values, fold change values, and regulation status for statistically significant metabolites in CU compared to nonusers (Table S1); VIP scores of metabolites (Table S2); and pathway enrichment analysis results based on MetaboAnalyst and CPDB (Table S3 and Table S4, respectively) (PDF)
