# Supplementary material for: Metabolic Profile of Male Cannabis Users and Estimation of Candidate Biomarkers
Source: Chem Res Toxicol. 2025 Oct 10;38(10):1762–70. doi: 10.1021/acs.chemrestox.5c00274 (PMC12541800; doi:10.1021/acs.chemrestox.5c00274)
Supplement: Supplementary file 1 [file tx5c00274_si_001.pdf]

Supporting Information

Metabolic profile of male cannabis users and estimation of candidate biomarkers

Esin Oz[1], Merve Kasikci[2], İbrahim Celik[3], Mukaddes Gurler[1,4*]

[1]Department of Medical Biochemistry, Faculty of Medicine, Hacettepe University, Ankara, Türkiye

[2]Department of Biostatistics, Faculty of Medicine, Hacettepe University, Ankara, Türkiye

[3] Faculty of Medicine, Hacettepe University, Ankara, Türkiye

[4]Department of Medical Biochemistry and Forensic Medicine, Faculty of Medicine, Hacettepe University, Ankara, Türkiye

* Corresponding Author

Supporting Information – Table of Contents

1. P-values, fold change values, and regulation status for statistically significant metabolites in cannabis users compared to non-users **Page S3-S6**
2. VIP scores of metabolites **Page S7-S8**
3. Results of pathway enrichment analysis conducted on MetaboAnalyst based on the KEGG database **Page S9**
4. Results of pathway enrichment analysis conducted on CPDB based on multiple databases **Page S10-S11**

P-values, fold change values, and regulation status for statistically significant metabolites are presented in Table S1. Out of over 200 metabolites detected in cannabis user samples, 92 with p-values < 0.05 were identified for further analysis, including 38 that decreased and 54 that increased compared to non-users.

Table S1. Results for metabolites that differed significantly between groups.

No	KEGG ID	KEGG Link	Name	p.value	FDR	Fold Change	UP/ DOWN
1	C09640	https://www.genome.jp/entry/C09640	(-)-Salsoline	<0.001	<0.001	6.288	UP
2	C16613	https://www.genome.jp/entry/C16613	6-Thiourate	<0.001	<0.001	8.666	UP
3	C01211	https://www.genome.jp/entry/C01211	Procollagen 5-hydroxy-L-lysine	<0.001	<0.001	32.194	UP
4	C00120	https://www.genome.jp/entry/C00120	Biotin	<0.001	0.001	3.165	DOWN
5	C03087	https://www.genome.jp/entry/C03087	5-Acetamidovalerate	<0.001	<0.001	23.151	UP
6	C01449	https://www.genome.jp/entry/C01449	Queuine	<0.001	<0.001	2.404	DOWN
7	C05933	https://www.genome.jp/entry/C05933	N(omega)-Hydroxyarginine	<0.001	0.002	3.115	DOWN
8	C05401	https://www.genome.jp/entry/C05401	Galactosylglycerol	<0.001	<0.001	2.075	DOWN
9	C00670	https://www.genome.jp/entry/C00670	Glycerophosphocholine	<0.001	0.001	7.042	DOWN
10	C01595	https://www.genome.jp/entry/C01595	Linoleic acid	<0.001	<0.001	3.788	DOWN
11	C00957	https://www.genome.jp/entry/C00957	3-Mercaptopyruvic acid	<0.001	<0.001	5.714	DOWN
12	C02218	https://www.genome.jp/entry/C02218	2-Aminoacrylic acid	<0.001	<0.001	6.828	UP
13	C02576	https://www.genome.jp/entry/C02576	Perillyl aldehyde	<0.001	<0.001	6.024	DOWN
14	C08012	https://www.genome.jp/entry/C08012	Levomethadyl Acetate	<0.001	<0.001	6.757	DOWN
15	C00093	https://www.genome.jp/entry/C00093	Glycerol 3-phosphate	<0.001	<0.001	3.861	DOWN
16	C00664	https://www.genome.jp/entry/C00664	5-Formiminotetrahydrofolic acid	<0.001	<0.001	5.859	UP
17	C05823	https://www.genome.jp/entry/C05823	3-Mercaptolactic acid	<0.001	<0.001	4.721	UP
18	C04441	https://www.genome.jp/entry/C04441	2-(3-Carboxy-3-aminopropyl)-L-histidine	<0.001	0.001	1.953	DOWN
19	C00086	https://www.genome.jp/entry/C00086	Urea	<0.001	<0.001	9.091	DOWN
20	C00390	https://www.genome.jp/entry/C00390	QH2	<0.001	0.001	2.532	DOWN
21	C00850	https://www.genome.jp/entry/C00850	Aryl sulfate; Phenol sulfate	<0.001	<0.001	3.817	DOWN
22	C03033	https://www.genome.jp/entry/C03033	3-Methoxy-4-hydroxyphenylglycol glucuronide	<0.001	<0.001	2.584	DOWN
23	C01031	https://www.genome.jp/entry/C01031	S-Formylglutathione	<0.001	<0.001	7.213	UP
24	C03406	https://www.genome.jp/entry/C03406	Argininosuccinic acid	<0.001	<0.001	3.247	DOWN
25	C06423	https://www.genome.jp/entry/C06423	Caprylic acid	<0.001	<0.001	5.952	DOWN

26	C02325	https://www.genome.jp/entry/C02325	Sinapyl alcohol	<0.001	<0.001	7.663	UP
27	C01152	https://www.genome.jp/entry/C01152	1-Methylhistidine	<0.001	0.001	7.246	DOWN
28	C07535	https://www.genome.jp/entry/C07535	Benzo[a]pyrene	<0.001	0.001	2.688	DOWN
29	C16014	https://www.genome.jp/entry/C16014	cis-stilbene oxide	<0.001	<0.001	3.399	UP
30	C05504	https://www.genome.jp/entry/C05504	Estriol-16-Glucuronide	0.001	0.003	2.77	DOWN
31	C00037	https://www.genome.jp/entry/C00037	Glycine	<0.001	<0.001	3.8	UP
32	C00245	https://www.genome.jp/entry/C00245	Taurine	<0.001	<0.001	3.323	UP
33	C00864	https://www.genome.jp/entry/C00864	Pantothenic acid	<0.001	<0.001	2.41	UP
34	C05552	https://www.genome.jp/entry/C05552	Biocytin	<0.001	<0.001	5.917	DOWN
35	C00353	https://www.genome.jp/entry/C00353	Geranylgeranyl-PP	<0.001	0.001	3.968	DOWN
36	C05839	https://www.genome.jp/entry/C05839	cis-beta-D-Glucosyl-2-hydroxycinnamate	<0.001	<0.001	4.371	UP
37	C16015	https://www.genome.jp/entry/C16015	(+)-(1R,2R)-1,2-Diphenylethane-1,2-diol	<0.001	0.001	2.237	DOWN
38	C05985	https://www.genome.jp/entry/C05985	2-Propyn-1-al	<0.001	<0.001	4.717	DOWN
39	C05588	https://www.genome.jp/entry/C05588	Metanephrine	0.005	0.014	1.661	DOWN
40	C14852	https://www.genome.jp/entry/C14852	Benzo[a]pyrene-7,8-diol	0.006	0.015	2.427	DOWN
41	C02727	https://www.genome.jp/entry/C02727	N6-Acetyl-L-lysine	0.002	0.006	3.077	DOWN
42	C00409	https://www.genome.jp/entry/C00409	Methanethiol	<0.001	<0.001	3.861	DOWN
43	C03492	https://www.genome.jp/entry/C03492	D-4'-Phosphopantothenate	<0.001	0.001	2.173	UP
44	C06755	https://www.genome.jp/entry/C06755	Chloroacetic acid	<0.001	<0.001	4.454	UP
45	C00988	https://www.genome.jp/entry/C00988	Phosphoglycolic acid	<0.001	0.002	13.158	DOWN
46	C16632	https://www.genome.jp/entry/C16632	alpha-Fluoro-beta-alanine	<0.001	<0.001	8.599	UP
47	C03078	https://www.genome.jp/entry/C03078	4-Guanidinobutanamide	<0.001	<0.001	3.708	UP
48	C01119	https://www.genome.jp/entry/C01119	Oxidized dithiothreitol	0.001	0.004	6.41	DOWN
49	C00327	https://www.genome.jp/entry/C00327	Citrulline	<0.001	0.001	6.095	UP
50	C03413	https://www.genome.jp/entry/C03413	N1,N12-Diacetylspermine	0.028	0.054	3.002	DOWN
51	C00058	https://www.genome.jp/entry/C00058	Formic acid	<0.001	<0.001	3.096	DOWN
52	C02373	https://www.genome.jp/entry/C02373	3a,7b,12a-Trihydroxy-5a-Cholanoic acid	<0.001	0.002	2.294	DOWN
53	C03340	https://www.genome.jp/entry/C03340	L-2,3-Dihydrodipicolinate	<0.001	<0.001	2.432	UP
54	C08261	https://www.genome.jp/entry/C08261	Azelaic acid	<0.001	0.001	3.831	DOWN
55	C00440	https://www.genome.jp/entry/C00440	5-Methyltetrahydrofolic acid	0.016	0.034	1.65	DOWN
56	C16662	https://www.genome.jp/entry/C16662	L-alpha-Acetyl-N,N-dinormethadol	0.014	0.030	4.098	DOWN

57	C04352	https://www.genome.jp/entry/C04352	4'-Phosphopantothenoylcysteine	<0.001	0.001	2.159	UP
58	C06350	https://www.genome.jp/entry/C06350	Tetrahydropapaveroline; (R,S)-Norlaudanosoline; Norlaudanosoline	0.027	0.052	2.762	DOWN
59	C00621	https://www.genome.jp/entry/C00621	Dolichyl diphosphate	<0.001	0.001	2.042	UP
60	C15650	https://www.genome.jp/entry/C15650	5-(Methylthio)-2,3-dioxopentyl phosphate	0.001	0.002	2.363	UP
61	C03356	https://www.genome.jp/entry/C03356	3-Phospho-D-erythronate	<0.001	<0.001	2.52	UP
62	C00073	https://www.genome.jp/entry/C00073	L-Methionine	0.047	0.081	1.506	DOWN
63	C01620	https://www.genome.jp/entry/C01620	Threonic acid	<0.001	<0.001	2.154	UP
64	C02637	https://www.genome.jp/entry/C02637	3-Dehydroshikimate	<0.001	0.001	2.212	UP
65	C06206	https://www.genome.jp/entry/C06206	Benzoyl phosphate	0.002	0.006	2.428	UP
66	C14873	https://www.genome.jp/entry/C14873	Thiodiacetic acid sulfoxide	0.004	0.010	1.861	UP
67	C00027	https://www.genome.jp/entry/C00027	Hydrogen peroxide	<0.001	0.001	2.075	UP
68	C00460	https://www.genome.jp/entry/C00460	Deoxyuridine triphosphate	<0.001	<0.001	1.917	UP
69	C05379	https://www.genome.jp/entry/C05379	Oxalosuccinic acid	0.001	0.003	1.978	UP
70	C04409	https://www.genome.jp/entry/C04409	2-Amino-3-carboxymuconic acid semialdehyde	0.004	0.010	2.168	UP
71	C00506	https://www.genome.jp/entry/C00506	Cysteic acid	0.003	0.008	2.102	UP
72	C05403	https://www.genome.jp/entry/C05403	3'-Ketolactose	0.001	0.003	2.258	UP
73	C00322	https://www.genome.jp/entry/C00322	Oxoadipic acid	0.002	0.006	1.927	UP
74	C05335	https://www.genome.jp/entry/C05335	Selenomethionine	0.001	0.003	3.084	UP
75	C01345	https://www.genome.jp/entry/C01345	dITP	0.004	0.012	2.419	UP
76	C14872	https://www.genome.jp/entry/C14872	Thiodiacetic acid	<0.001	0.002	2.173	UP
77	C00155	https://www.genome.jp/entry/C00155	L-Homocysteine	0.02	0.042	1.651	UP
78	C03415	https://www.genome.jp/entry/C03415	N2-Succinyl-L-ornithine	0.021	0.043	1.721	UP
79	C00603	https://www.genome.jp/entry/C00603	(S)-Ureidoglycolic acid	0.017	0.037	1.655	UP
80	C00387	https://www.genome.jp/entry/C00387	Guanosine	0.038	0.069	1.648	UP
81	C14854	https://www.genome.jp/entry/C14854	9-Hydroxybenzo[a]pyrene-4,5-oxide	0.042	0.075	1.662	UP
82	C05841	https://www.genome.jp/entry/C05841	Nicotinate D-ribonucleoside	<0.001	0.002	1.931	UP
83	C00053	https://www.genome.jp/entry/C00053	Phosphoadenosine phosphosulfate	0.041	0.073	1.794	UP
84	C14875	https://www.genome.jp/entry/C14875	S-(2-Hydroxyethyl)glutathione	0.022	0.045	2.015	UP
85	C03410	https://www.genome.jp/entry/C03410	N-Glycolylneuraminic acid	0.014	0.030	1.56	UP
86	C02220	https://www.genome.jp/entry/C02220	2-Aminomuconic acid	0.025	0.049	1.671	UP

87	C11132	https://www.genome.jp/entry/C11132	2-Methoxyestrone 3-glucuronide	0.039	0.071	1.356	UP
88	C00169	https://www.genome.jp/entry/C00169	Carbamoyl phosphate	0.006	0.016	1.135	UP
89	C02470	https://www.genome.jp/entry/C02470	Xanthurenic acid	0.026	0.051	1.588	UP
90	C06429	https://www.genome.jp/entry/C06429	Docosahexaenoic acid	0.021	0.042	4.787	UP
91	C01044	https://www.genome.jp/entry/C01044	N-Formyl-L-aspartate	0.039	0.070	1.853	UP
92	C01417	https://www.genome.jp/entry/C01417	Cyanate	0.008	0.020	1.193	UP

Table S2. VIP scores of metabolites.

No	KEGG ID	Name	VIP score
1	C09640	(-)-Salsoline	1.439
2	C16613	6-Thiourate	1.427
3	C01211	Procollagen 5-hydroxy-L-lysine	1.419
4	C00120	Biotin	1.415
5	C03087	5-Acetamidovalerate	1.395
6	C01449	Queuine	1.392
7	C05933	N(omega)-Hydroxyarginine	1.323
8	C05401	Galactosylglycerol	1.311
9	C00670	Glycerophosphocholine	1.298
10	C01595	Linoleic acid	1.298
11	C00957	3-Mercaptopyruvic acid	1.295
12	C02218	2-Aminoacrylic acid	1.286
13	C02576	Perillyl aldehyde	1.283
14	C08012	Levomethadyl Acetate	1.274
15	C00093	Glycerol 3-phosphate	1.250
16	C00664	5-Formiminotetrahydrofolic acid	1.249
17	C05823	3-Mercaptolactic acid	1.245
18	C04441	2-(3-Carboxy-3-aminopropyl)-L-histidine	1.245
19	C00086	Urea	1.236
20	C00390	QH2	1.230
21	C00850	Aryl sulfate; Phenol sulfate	1.219
22	C03033	3-Methoxy-4-hydroxyphenylglycol glucuronide	1.214
23	C01031	S-Formylglutathione	1.212
24	C03406	Argininosuccinic acid	1.207
25	C06423	Caprylic acid	1.195
26	C02325	Sinapyl alcohol	1.189
27	C01152	1-Methylhistidine	1.158
28	C07535	Benzo[a]pyrene	1.147
29	C16014	cis-stilbene oxide	1.140
30	C05504	Estriol-16-Glucuronide	1.135
31	C00037	Glycine	1.126
32	C00245	Taurine	1.113
33	C00864	Pantothenic acid	1.097
34	C05552	Biocytin	1.097
35	C00353	Geranylgeranyl-PP	1.095
36	C05839	cis-beta-D-Glucosyl-2-hydroxycinnamate	1.084
37	C16015	(+)-(1R,2R)-1,2-Diphenylethane-1,2-diol	1.070
38	C05985	2-Propyn-1-al	1.063
39	C05588	Metanephrine	1.046
40	C14852	Benzo[a]pyrene-7,8-diol	1.042
41	C02727	N6-Acetyl-L-lysine	1.041
42	C00409	Methanethiol	1.021
43	C03492	D-4'-Phosphopantothenate	1.011
44	C06755	Chloroacetic acid	0.990
45	C00988	Phosphoglycolic acid	0.980

46	C16632	alpha-Fluoro-beta-alanine	0.966
47	C03078	4-Guanidinobutanamide	0.959
48	C01119	Oxidized dithiothreitol	0.958
49	C00327	Citrulline	0.955
50	C03413	N1,N12-Diacetylspermine	0.949
51	C00058	Formic acid	0.948
52	C02373	3a,7b,12a-Trihydroxy-5a-Cholanoic acid	0.927
53	C03340	L-2,3-Dihydrodipicolinate	0.920
54	C08261	Azelaic acid	0.906
55	C00440	5-Methyltetrahydrofolic acid	0.886
56	C16662	L-alpha-Acetyl-N,N-dinormethadol	0.873
57	C04352	4'-Phosphopantothenoylcysteine	0.862
58	C06350	Tetrahydropapaveroline; (R,S)-Norlaudanosoline; Norlaudanosoline	0.855
59	C00621	Dolichyl diphosphate	0.853
60	C15650	5-(Methylthio)-2,3-dioxopentyl phosphate	0.852
61	C03356	3-Phospho-D-erythronate	0.847
62	C00073	L-Methionine	0.817
63	C01620	Threonic acid	0.816
64	C02637	3-Dehydroshikimate	0.812
65	C06206	Benzoyl phosphate	0.797
66	C14873	Thiodiacetic acid sulfoxide	0.781
67	C00027	Hydrogen peroxide	0.764
68	C00460	Deoxyuridine triphosphate	0.747
69	C05379	Oxalosuccinic acid	0.738
70	C04409	2-Amino-3-carboxymuconic acid semialdehyde	0.733
71	C00506	Cysteic acid	0.720
72	C05403	3'-Ketolactose	0.716
73	C00322	Oxoadipic acid	0.660
74	C05335	Selenomethionine	0.645
75	C01345	dITP	0.640
76	C14872	Thiodiacetic acid	0.636
77	C00155	L-Homocysteine	0.615
78	C03415	N2-Succinyl-L-ornithine	0.613
79	C00603	(S)-Ureidoglycolic acid	0.602
80	C00387	Guanosine	0.578
81	C14854	9-Hydroxybenzo[a]pyrene-4,5-oxide	0.577
82	C05841	Nicotinate D-ribonucleoside	0.575
83	C00053	Phosphoadenosine phosphosulfate	0.574
84	C14875	S-(2-Hydroxyethyl)glutathione	0.572
85	C03410	N-Glycolylneuraminic acid	0.496
86	C02220	2-Aminomuconic acid	0.484
87	C11132	2-Methoxyestrone 3-glucuronide	0.470
88	C00169	Carbamoyl phosphate	0.467
89	C02470	Xanthurenic acid	0.465
90	C06429	Docosahexaenoic acid	0.382
91	C01044	N-Formyl-L-aspartate	0.377
92	C01417	Cyanate	0.335

Pathway enrichment analysis was also conducted on CPDB since this software simultaneously considers several databases in addition to KEGG. The results of enrichment analysis for Metaboanalyst and CPDB are shown in Tables S3 and S4.

Table S3. Results of pathway enrichment analysis conducted on MetaboAnalyst based on KEGG database.

Pathway	Total	Hits	p-value
Biotin metabolism	10	2	0.012
Cysteine and methionine metabolism	33	3	0.019
Arginine biosynthesis	14	2	0.024
Pantothenate and CoA biosynthesis	20	2	0.046
Linoleic acid metabolism	5	1	0.085
Glycerophospholipid metabolism	36	2	0.130
Taurine and hypotaurine metabolism	8	1	0.132
Sulfur metabolism	8	1	0.132
One carbon pool by folate	9	1	0.148
Primary bile acid biosynthesis	46	2	0.192
Glycerolipid metabolism	16	1	0.248
Ubiquinone and other terpenoid-quinone biosynthesis	18	1	0.274
Terpenoid backbone biosynthesis	18	1	0.274
Ether lipid metabolism	20	1	0.300
Metabolism of xenobiotics by cytochrome P450	68	2	0.337
Galactose metabolism	27	1	0.382
Alanine, aspartate and glutamate metabolism	28	1	0.393
Glutathione metabolism	28	1	0.393
Lipoic acid metabolism	28	1	0.393
Lysine degradation	30	1	0.415
Glyoxylate and dicarboxylate metabolism	31	1	0.425
Porphyrin metabolism	31	1	0.425
Glycine, serine and threonine metabolism	33	1	0.446
Arginine and proline metabolism	36	1	0.475
Biosynthesis of unsaturated fatty acids	36	1	0.475
Drug metabolism - other enzymes	39	1	0.503
Fatty acid biosynthesis	47	1	0.570
Drug metabolism - cytochrome P450	55	1	0.629
Purine metabolism	70	1	0.719
Steroid hormone biosynthesis	87	1	0.795

Table S4. Results of pathway enrichment analysis conducted on CPDB based on multiple databases.

Pathway	Total	Hits	p-value	Database
Methionine and cysteine metabolism	80	5	0.001	EHMN
Amino acid conjugation	6	2	0.001	Wikipathways
Lysine degradation - Homo sapiens (human)	50	4	0.001	KEGG
Hyperornithinemia with gyrate atrophy (HOGA)	52	4	0.001	SMPDB
Creatine deficiency, guanidinoacetate methyltransferase deficiency	52	4	0.001	SMPDB
L-arginine:glycine amidinotransferase deficiency	52	4	0.001	SMPDB
Hyperornithinemia-hyperammonemia-homocitrullinuria [HHH-syndrome]	52	4	0.001	SMPDB
Guanidinoacetate Methyltransferase Deficiency (GAMT Deficiency)	52	4	0.001	SMPDB
Prolinemia Type II	52	4	0.001	SMPDB
Prolidase Deficiency (PD)	52	4	0.001	SMPDB
Arginine and Proline Metabolism	52	4	0.001	SMPDB
Hyperprolinemia Type I	52	4	0.001	SMPDB
Hyperprolinemia Type II	52	4	0.001	SMPDB
Ornithine Aminotransferase Deficiency (OAT Deficiency)	52	4	0.001	SMPDB
Arginine: Glycine Amidinotransferase Deficiency (AGAT Deficiency)	52	4	0.001	SMPDB
Metabolism of vitamins and cofactors	188	6	0.001	Reactome
Metabolism of water-soluble vitamins and cofactors	125	5	0.001	Reactome
Biotin Metabolism	7	2	0.002	SMPDB
Multiple carboxylase deficiency, neonatal or early onset form	7	2	0.002	SMPDB
Biotinidase Deficiency	7	2	0.002	SMPDB
Biotin metabolism - Homo sapiens (human)	28	3	0.002	KEGG
SLC-mediated transmembrane transport	164	6	0.002	Reactome
Hydrolysis of LPC	9	2	0.002	Reactome
Glycerophospholipid catabolism	11	2	0.002	Reactome
Cysteine and methionine metabolism - Homo sapiens (human)	63	4	0.002	KEGG
Biotin transport and metabolism	9	2	0.003	Reactome
Arginine Proline metabolism	68	4	0.003	INOH
Degradation of cysteine and homocysteine	40	3	0.003	Reactome
Choline metabolism in cancer - Homo sapiens (human)	11	2	0.004	KEGG
Vitamin H (biotin) metabolism	11	2	0.004	EHMN
Metabolism of amino acids and derivatives	286	7	0.004	Reactome
Vitamin digestion and absorption - Homo sapiens (human)	39	3	0.004	KEGG
Biochemical Pathways Part I	469	10	0.005	Wikipathways
Coenzyme A biosynthesis	13	2	0.005	Reactome
coenzyme A biosynthesis	13	2	0.005	HumanCyc
glutathione-mediated detoxification	14	2	0.006	HumanCyc
ABC transporters - Homo sapiens (human)	138	5	0.006	KEGG
Transport of small molecules	219	6	0.006	Reactome
Methionine Cysteine metabolism	45	3	0.007	INOH
Glycerophospholipid metabolism	95	4	0.009	EHMN
Pyruvate Carboxylase Deficiency	17	2	0.009	SMPDB
Primary Hyperoxaluria Type I	17	2	0.009	SMPDB
Lactic Acidemia	17	2	0.009	SMPDB
Alanine Metabolism	17	2	0.009	SMPDB
urea cycle	17	2	0.009	HumanCyc
Vitamin B5 (pantothenate) metabolism	17	2	0.009	Reactome
Metabolism	1411	15	0.010	Reactome
Sulfur amino acid metabolism	67	3	0.011	Reactome
Fatty Acid Biosynthesis	33	2	0.012	SMPDB
Methionine metabolism leading to sulfur amino acids and related disorders	21	2	0.012	Wikipathways
Amino Acid metabolism	108	4	0.013	Wikipathways
Recycling of bile acids and salts	20	2	0.013	Reactome
Pantothenate and CoA Biosynthesis	21	2	0.014	SMPDB
Vitamin B5 - CoA biosynthesis from pantothenate	21	2	0.014	EHMN

Arginine biosynthesis - Homo sapiens (human)	23	2	0.017	KEGG
Urea cycle	23	2	0.017	Reactome
Transport of vitamins, nucleosides, and related molecules	63	3	0.017	Reactome
Disorders of Folate Metabolism and Transport	26	2	0.020	Wikipathways
PI Metabolism	29	2	0.020	Reactome
Glycine Serine metabolism	68	3	0.021	INOH
Beta-mercaptolactate-cysteine disulfiduria	26	2	0.021	SMPDB
Cysteine Metabolism	26	2	0.021	SMPDB
Cystinosis, ocular nonnephropathic	26	2	0.021	SMPDB
Urea cycle and metabolism of arginine, proline, glutamate, aspartate and asparagine	125	4	0.022	EHMN
Argininemia	27	2	0.023	SMPDB
Citrullinemia Type I	27	2	0.023	SMPDB
Carbamoyl Phosphate Synthetase Deficiency	27	2	0.023	SMPDB
Argininosuccinic Aciduria	27	2	0.023	SMPDB
Urea Cycle	27	2	0.023	SMPDB
Ornithine Transcarbamylase Deficiency (OTC Deficiency)	27	2	0.023	SMPDB
Transport of bile salts and organic acids, metal ions and amine compounds	79	3	0.023	Reactome
Na+/Cl- dependent neurotransmitter transporters	32	2	0.024	Reactome
Glutathione conjugation	31	2	0.024	Reactome
Amino acid transport across the plasma membrane	32	2	0.026	Reactome
Ammonia Recycling	29	2	0.026	SMPDB
Pantothenate and CoA biosynthesis - Homo sapiens (human)	30	2	0.028	KEGG
Trans-sulfuration and one-carbon metabolism	33	2	0.029	Wikipathways
Urea cycle and metabolism of amino groups	32	2	0.029	Wikipathways
Pyruvate dehydrogenase deficiency (E3)	33	2	0.031	SMPDB
Pyruvate dehydrogenase deficiency (E2)	33	2	0.031	SMPDB
2-ketoglutarate dehydrogenase complex deficiency	33	2	0.031	SMPDB
Mitochondrial complex II deficiency	33	2	0.031	SMPDB
Fumarase deficiency	33	2	0.031	SMPDB
Congenital lactic acidosis	33	2	0.031	SMPDB
Citric Acid Cycle	33	2	0.031	SMPDB
beta-Alanine metabolism - Homo sapiens (human)	32	2	0.031	KEGG
triacylglycerol degradation	54	2	0.031	HumanCyc
Sulfur metabolism - Homo sapiens (human)	33	2	0.033	KEGG
Carnosinuria, carnosinemia	34	2	0.033	SMPDB
Ureidopropionase deficiency	34	2	0.033	SMPDB
GABA-Transaminase Deficiency	34	2	0.033	SMPDB
Beta-Alanine Metabolism	34	2	0.033	SMPDB
phospholipases	55	2	0.035	HumanCyc
sphingomyelin metabolism/ceramide salvage	55	2	0.035	HumanCyc
Vitamin B9 (folate) metabolism	35	2	0.037	EHMN
Citrate cycle	35	2	0.037	INOH
Lysine degradation	36	2	0.039	INOH
Glycerolipid metabolism - Homo sapiens (human)	38	2	0.043	KEGG
The oncogenic action of 2-hydroxyglutarate	40	2	0.045	SMPDB
Histidine Metabolism	40	2	0.045	SMPDB
Histidinemia	40	2	0.045	SMPDB
the visual cycle I (vertebrates)	58	2	0.045	HumanCyc
The oncogenic action of Succinate	41	2	0.047	SMPDB
The oncogenic action of L-2-hydroxyglutarate in Hydroxygluaricaciduria	41	2	0.047	SMPDB
The oncogenic action of D-2-hydroxyglutarate in Hydroxygluaricaciduria	41	2	0.047	SMPDB
sphingosine and sphingosine-1-phosphate metabolism	59	2	0.047	HumanCyc
The oncogenic action of Fumarate	42	2	0.049	SMPDB
